# Engineering Nanoplatform for Combined Cancer Therapeutics via Complementary Autophagy Inhibition

**DOI:** 10.3390/ijms23020657

**Published:** 2022-01-07

**Authors:** Xuan Wang, Yunhao Li, Jianqing Lu, Xiongwei Deng, Yan Wu

**Affiliations:** 1 CAS Key Laboratory for Biomedical Effects of Nanomaterials and Nanosafety, CAS Center for Excellence in Nanoscience, National Center for Nanoscience and Technology, Beijing 100190, China; wangxuan@nanoctr.cn (X.W.); lujq@nanoctr.cn (J.L.); 2 University of Chinese Academy of Sciences, Beijing 100049, China; 3 Department of General Surgery, Peking Union Medical College Hospital, Peking Union Medical College, Chinese Academy of Medical Sciences, Beijing 100730, China; lyh2423@126.com

**Keywords:** autophagy inhibition, tumor treatment, nanoparticles

## Abstract

Despite advances in the development of tumor treatments, mortality from cancer continues to increase. Nanotechnology is expected to provide an innovative anti-cancer therapy, to combat challenges such as multidrug resistance and tumor recurrence. Nevertheless, tumors can greatly rely on autophagy as an alternative source for metabolites, and which desensitizes cancer cells to therapeutic stress, hindering the success of any current treatment paradigm. Autophagy is a conserved process by which cells turn over their own constituents to maintain cellular homeostasis. The multistep autophagic pathway provides potentially druggable targets to inhibit pro-survival autophagy under various therapeutic stimuli. In this review, we focus on autophagy inhibition based on functional nanoplatforms, which may be a potential strategy to increase therapeutic sensitivity in combinational cancer therapies, including chemotherapy, radiotherapy, phototherapy, sonodynamic therapy, and immunotherapy.

## 1. Introduction

Cancer, a leading cause of morbidity and mortality, is a disease that is still an impossible burden on humans, a conclusion that can be derived from its diagnosis, management, and care [1,2]. Classically, cancer treatment relying on chemo-drugs suffered from major drawbacks, such as its indiscriminate toxicity and side effects. Cancer therapeutic agents have been approved for clinical usage, including small molecules, antibodies, and nanodrugs [3,4,5]. Over the recent decades, nanodrugs exhibited enhanced permeability and retention (EPR) effects due to their nanoscale size of 10~200 nm diameter, and are widely used in cancer therapy for their advantages, including improved side effects, extended circulation time, reduced toxicity, and increased target distribution [6,7,8,9]. Despite significant advances in nanomedicine in enhancing the safety and potency of cancer therapy, there exists underlying therapeutic resistance. It can be putative that cancer cells have also developed protective mechanisms to skip cell apoptosis for continued survival upon exposure to therapeutic stimuli.

**Figure 1:**
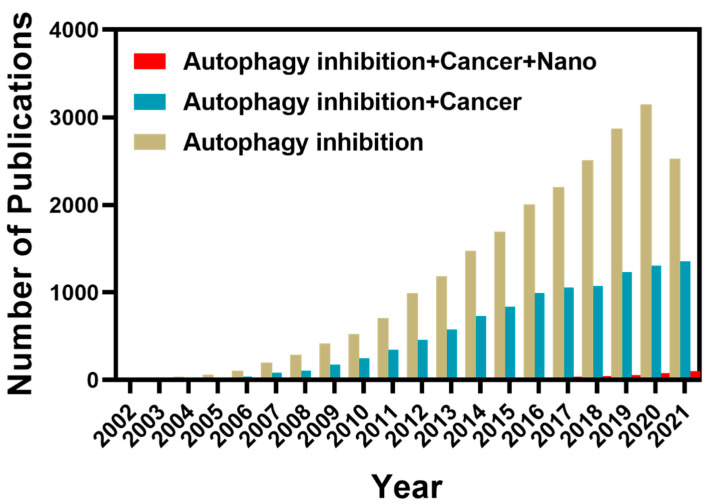
The publication record of Autophagy inhibition, Autophagy inhibition + Cancer therapy and Autophagy inhibition + Cancer therapy + Nano. (Source: Web of Science).

Autophagy [10,11,12] is a cellular self-degradation metabolism process and plays a predominant role in maintaining cellular homeostasis. Autophagic flux is a multistep process. In the initial step, autophagy-related protein 4 (Atg4) hydrolyzes the intracellular microtubule-associated protein light chain 3 (LC3) precursor to generate water-soluble LC3-I in the cytoplasm [13,14]. Subsequently, the synergistic action of Atg7 and the Atg12–Atg5–Atg16l complex could help LC3-I covalently bind to phosphatidyl ethanolamine (PE) to generate lipid-soluble LC3-II [15]. Then, the autophagosome membrane extends with LC3-II. Autophagosomes are formed as double-membraned vesicles to sequester cargo for delivery to the lysosome [16]. Finally, autolysosomes could be formed following the fusion of autophagosomes with lysosomes, which degrade the encapsulated materials to contribute to cell growth and maintain cell homeostasis. The increase of autolysosomes, the conversion of LC3-I to LC3-II, and the downregulation of the p62 protein are often used as markers of autophagy [17]. Nevertheless, there is a complex relationship between autophagy and tumor treatment [18]. Collective studies [19,20] have documented that autophagy plays an essential role in cancer cell survival, hindering the current treatment paradigm. Autophagy is additionally activated under different stress conditions, such as chemo-drugs, radiation, hyperthermia, and reactive oxygen species (ROS) [21,22,23]. Afterward, the tumor could greatly rely on autophagy to maintain an alternative source for metabolites, which desensitize cancer cells to therapeutic stress [24,25,26]. As shown in Figure 1, a universal search on Web of Science, using ‘‘autophagy inhibition’’ and ‘‘autophagy inhibition plus cancer therapy’’ as key words, shows increased studies having been published since 2007. Moreover, studies [27] in recent years have shown that strategies for rationally designing nanoparticles for targeted, multimodal delivery of therapeutic agents led to extraordinary toxic effects both in vivo and in vitro. As well, some reviews [14] mainly focus on the regulation of nanomaterial-mediated autophagy, bettering our understanding of the underlying mechanism by which nanotechnology regulates autophagy in cancer therapy. More attention should be paid to designing functional nanoplatform-mediated autophagy inhibition as an effective therapeutic tool for tumor treatment. Accordingly, nanoplatform-mediated autophagy inhibition for cancer therapy is an emerging area in the biomedical field. Based on the congenital advantages of nanoplatform [28], there is an urgent need for developing combined treatment with autophagy inhibition based on nanomedicine to increase cancer therapeutic sensitivity [10,29].

The multistep autophagic pathway provides potentially druggable targets to inhibit pro-survival autophagy under various therapeutic stimuli [30,31]. Classical autophagy inhibitors such as 3-methyladenine (3-MA), chloroquine (CQ), and bafilomycin A1 (BafA1) were used for clinical trials, which inhibit the initial or late stage of autophagy. 3-MA, a type of phosphoinositide 3-kinase (PI3K) inhibitor, is widely used to inhibit the initial stage of autophagy owing to their inhibitory effect on class III PI3K activity [32]. The most widely employed chemicals that inhibit the final stage of autophagy through increasing lysosomal pH to inhibit the activity of resident hydrolases include chloroquine (CQ), bafilomycin A1 (BafA1), and lysosomal protease inhibitor cocktails [12]. The mammalian target of rapamycin complex 1 (mTORC1) is activated by different stimuli that could be considered the characterized regulator of autophagy. mTORC1 negatively regulates autophagy by inhibiting two downstream proteins, UNC-51-like kinase 1 (ULK1) and vacuolar protein sorting 34 (Vps34) [33]. Although novel compounds such as SAR405 and ATG4B have been recently developed to specifically inhibit these ATG components, these drugs do not exclusively affect autophagy and, more importantly, they are not yet licensed for clinical trials [34]. Compared to small molecule inhibitors, RNA interference (RNAi) technology is a more temperate way to suppress the expression of autophagy-related proteins via silencing target genes [35,36]. Although the knockdown of proteins involved in the fusion between autophagosomes and lysosomes such as STX17 could be used at least in cell culture experiments, pharmacological inhibition is more kinetically controllable, and is the most frequently employed strategy for both in vitro and in vivo studies. In this way, further applications of RNAi technology were still limited due to the instability of gene delivery. An increasing number of studies [37,38] have been conducted regarding the co-delivery of the aforementioned autophagy inhibitors and other therapeutic agents based on nanoplatform. Along with the development of multifunctional nanoplatforms, however, there are intractable problems to cope with, including the low loading efficiency of multiple-drugs and uncontrolled drug release. Accordingly, there remains an imperative need to develop engineering nanoplatform via complementary autophagy inhibition for the enhancement of tumor treatment.

**Scheme 1:**
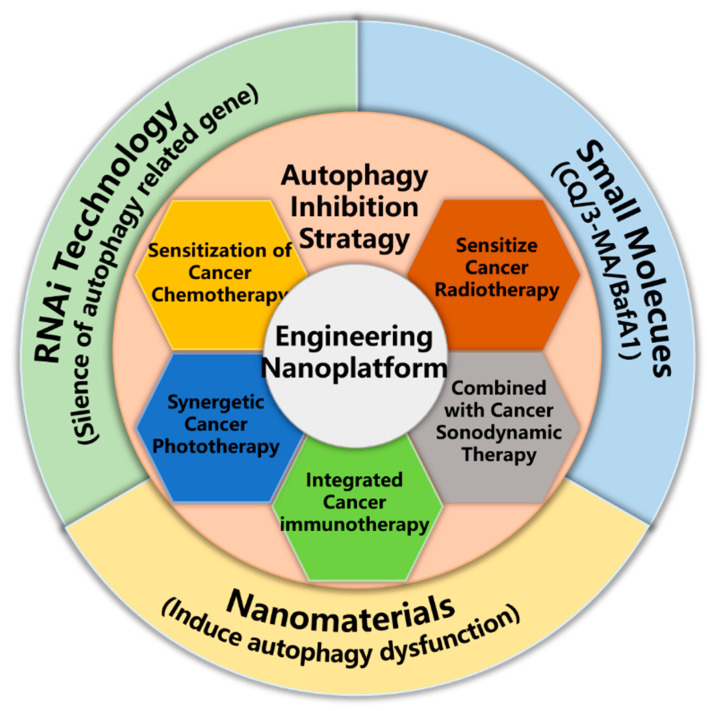
Schematic diagram of nanoplatform-mediated complementary autophagy inhibition strategy for combined cancer therapeutics.

In this review, we summarize works over the past 2–3 years which focus on autophagy inhibition involvement in cancer therapy in the context of nano-platforms as illustrated in Scheme 1. The inhibition of autophagy is crucial in compensating for the lack of a single therapeutic treatment. The advances in our knowledge of autophagy inhibition have driven focus on functionalized nanoplatforms through correctly combining autophagy inhibition with other therapeutic strategies for enhanced cancer therapy.

## 2. Exploitation of Nanoparticle Mediated Autophagy Inhibition Strategy for Cancer Therapy

The induction of autophagy in cancer cells occurred after long-term chemotherapy or in response to other therapeutic strategies [39,40]. Autophagy, as a protective self-degradative mechanism, could be viewed as having a pro-survival role in the resistance of cancer cells to various treatments. Given this, multidrug resistance and low therapeutic sensitive induced by autophagy could result in refractory cancer and tumor recurrence [41]. Accumulated data [42,43] have proposed that autophagy is activated in response to adverse stress and might be involved in the resistance to anticancer therapies such as chemotherapy, radiotherapy, phototherapy, and immunotherapy. Moreover, this process could mitigate therapeutic efficacy and contribute to resistance to treatments. Thus, autophagy inhibition can be an alternative strategy to re-sensitize resistance cancer cells and increase therapeutic sensitivity. Echoing this proposition, combinatorial therapy based on nanotechnological platforms is considered one of the most successful solutions in the clinic, shown in Table 1.

## 3. Sensitization of Cancer Chemotherapy

**Figure 2:**
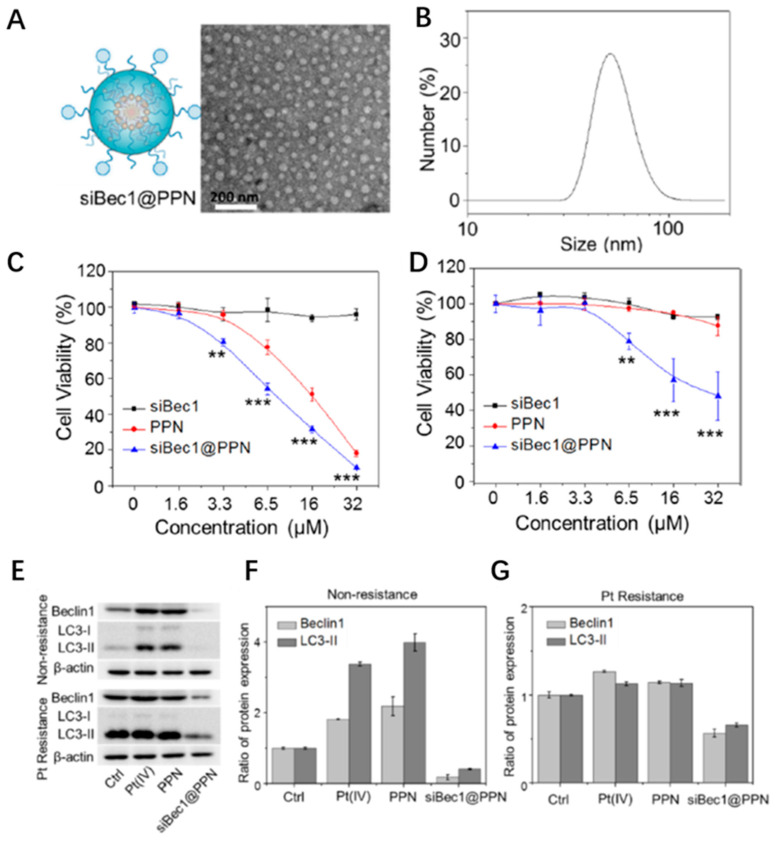
siBec1@PPN-enhanced apoptosis and reversed drug resistance by exploiting autophagy inhibition induced through silencing Beclin 1. (**A**) Schematic and TEM images of siBec1@PPN. (**B**) Size distribution of siBec1@PPN in pH 7.4 PBS buffer detected by DLS. Cell viability results of (**C**) normal A549 tumor cells and (**D**) Pt-resistant A549 tumor cells treated with siBec1, PPN, and siBec1@PPN for 48 h. siBec1@PPN-induced autophagy inhibition and intracellular GSH scavenge. *n* = 3; ** *p* < 0.01; *** *p* < 0.001. (**E**) Western blot of Beclin1 and LC3-II in A549 tumor cells or Pt-resistant A549 tumor cells treated with PBS (Ctrl), Pt(IV), PPN, and siBec1@PPN for 48 h. (**F**) The quantitative analysis results from panel (**G**) showing that autophagy is activated in both normal A549 cells and Pt-resistant A549 tumor cells(adapted from Reference [46] with permission from American Chemical Society. Copyright 2019, Nano letters).

Clinical chemotherapy is markedly hampered by multidrug resistance (MDR). MDR is inevitable following prolonged exposure to chemotherapeutic agents. Increasingly, recent studies have demonstrated that autophagy triggered by chemo-drugs may be involved in MDR, which facilitates the resistance of cancer cells to epirubicin, paclitaxel (PTX), or cisplatin. A prevailing approach to combat these thorny issues is to develop a nanotechnology-based co-delivery system incorporating a combination of autophagy inhibitors with chemotherapeutic agents. Autophagy inhibitors [56] such as chloroquine (CQ) and HCQ are widely used for co-delivery with chemo-drugs for the enhancement of chemotherapy. Compared to small molecular inhibitors [44], RNA interference technology is a more temperate way to silence the target gene. Meanwhile, a couple of recent studies have documented that the inhibition of autophagy via the genetic silencing of ATGs (such as Beclin1, ATG7, ATG5) is highly anticipated. In response to cisplatin, A549 cells exhibited Beclin1 (an autophagy-inducing peptide)-mediated protective autophagy, exemplified in a study by Lin [44] et al. Notably, it might be possible to inhibit autophagy through silencing Beclin1 via small interfere RNA. They constructed a novel self-assembled nano-prodrug platform (siBec1@PPN) consisting of Pt (IV)-peptide-bis(pyrene), DSPE-PEG, cRGD-modified DSPE-PEG, and Beclin1 siRNA. In the presence of glutathione (GSH), siBec1@PPN rapidly released active platinum ions (Pt (II)), which demonstrated negligible side effects in vivo. Meanwhile, Pt (IV)-peptide-bis(pyrene) acted as cationic carriers to deliver Beclin1 siRNA into the cytoplasm, thereby inhibiting autophagy via downregulating Beclin1 expression. As shown in Figure 2, deciphering the novel nanoplatform siBec1@PPN-mediated autophagy inhibition resulted in increased cytotoxicity of cisplatin in Pt-resistant A549 cells. Similar studies were reported by Gong’s group [45] for the inhibition of autophagy by genetic silencing of ATG7. siATG7 delivery in a crosslinked redox-sensitive polypeptide micellar system based on a lipoic acid (LA) conjugated with peptide 6 (CL, cell-penetrating peptide), which can effectively silence the ATG7 gene, suppressed DTX- induced autophagy, and exhibited improved anticancer effects. Analogously, Zhang [46] et al. developed a multifunctional micelle vehicle to encapsulate siATG7 and DTX. Subsequently, autophagy was inhibited by downregulating the expression of the ATG7 gene, which improved outcomes underlying the DTX treatment in pancreatic cancer. Nevertheless, microRNAs have also drawn increased attention for post-transcriptionally regulating gene expression. In particular, miR-101 suppressed autophagy induced by etoposide and rapamycin through the knockdown of three genes, STMN1, RAB5A, and ATG4D. Given this, Xin [57] and his colleagues established a carrier-free PTX nanocrystal (PNs), which was employed as a scaffold for the efficient intracellular delivery of miR-101 via a non-lysosomal pathway. It first demonstrated that the delivered miR-101 sensitizes cancer cells to the cytotoxic PNs by inhibiting autophagy, ultimately achieving a synergistic treatment of cancer.

## 4. Sensitize Cancer Radiotherapy

**Figure 3:**
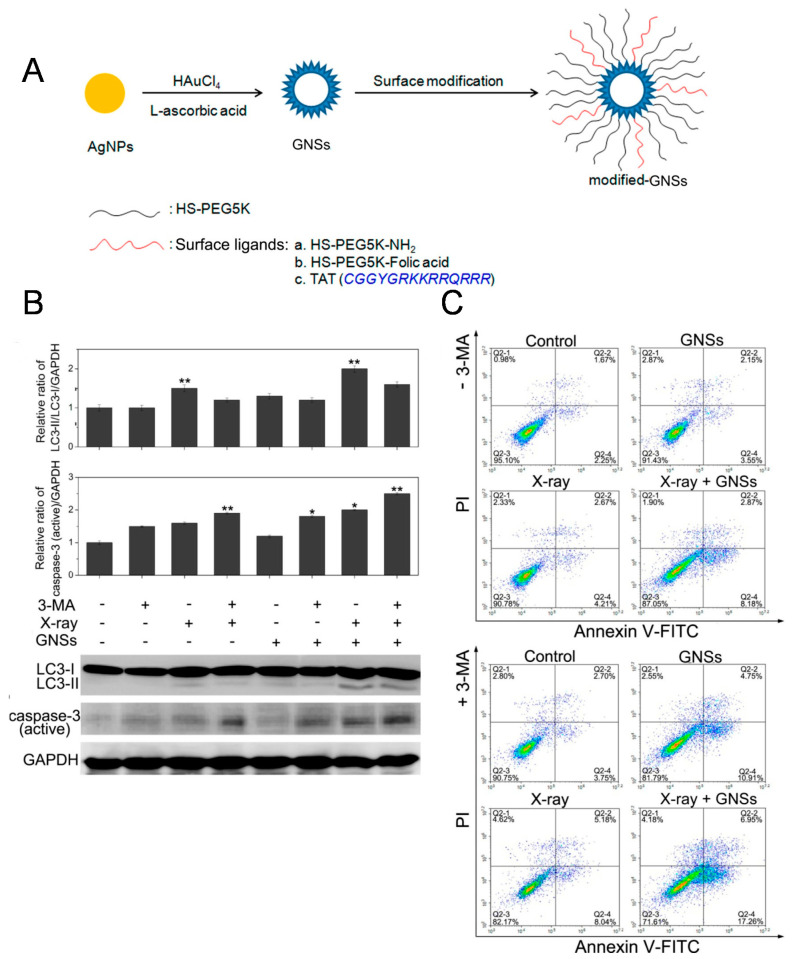
Enhancing RT for therapeutic resistance by combination with autophagy inhibitor 3-MA. (**A**) Schematic illustrating the preparation of PEG-modified GNSs. (**B**) Effect of 3-MA, X-ray, and GNSs on the expression level of LC3-I, LC3-II, and caspase-3 (active) in KB cells. The relative ratio of LC3-II/LC-I and caspase-3 (active) were quantified by integrated optical densitometry (IOD). *n* = 3; * *p* < 0.05; ** *p* < 0.01. GAPDH served as a loading control. (**C**) Effect of 3-MA on the apoptosis ratios of KB cells treated with culture medium (control), X-ray, GNSs, or X-ray + GNSs using Annexin V-FITC/PI staining (adapted from Reference [59] with permission from American Chemical Society. Copyright 2017, ACS Applied Materials & Interfaces).

Clinically, radiotherapy (RT) is one of the conventional approaches for cancer therapy. A prominent challenge for RT remains that radioresistant hallmarks enable cancer cells to escape the radiation-caused cell damages, resulting in the disappointing efficacy of RT. After exposure to radiation, tumor cells activate the self-repair mechanism to elicit radioresistance. Mounting studies [58] have shown that autophagy is one such biologic process involved in radioresistance. DNA damage caused by RT could trigger autophagy in response, which might be a reason for the therapeutic tolerance of RT. Some previous studies have demonstrated that gold nanoparticles and silver nanoparticles can be defined as autophagy activators. Recently, iron oxide nanoparticles have been reported to induce autophagy dysfunction by blocking autophagy flux. A new type of nanoparticle—Fe_3_O_4_@Ag, which has the characteristic of radiation enhancement by decreasing the cytoprotective autophagy of cells—was reported by Zhang [47] et al. Ma [59] et al. used a radiosensitizer based on gold nanoparticles (GNSs). However, cytoprotective autophagy was activated, hindering the favorable outcome and decreasing the efficacy of RT. A combination with autophagy inhibitor 3-MA was observed to significantly enhance the apoptosis of cancer cells, shown in Figure 3. Between them, a useful strategy for sensitizing cancer radiotherapy could involve the appropriate combined use of an autophagy inhibitor and metal nanostructures.

## 5. Synergetic Cancer Phototherapy

**Figure 4:**
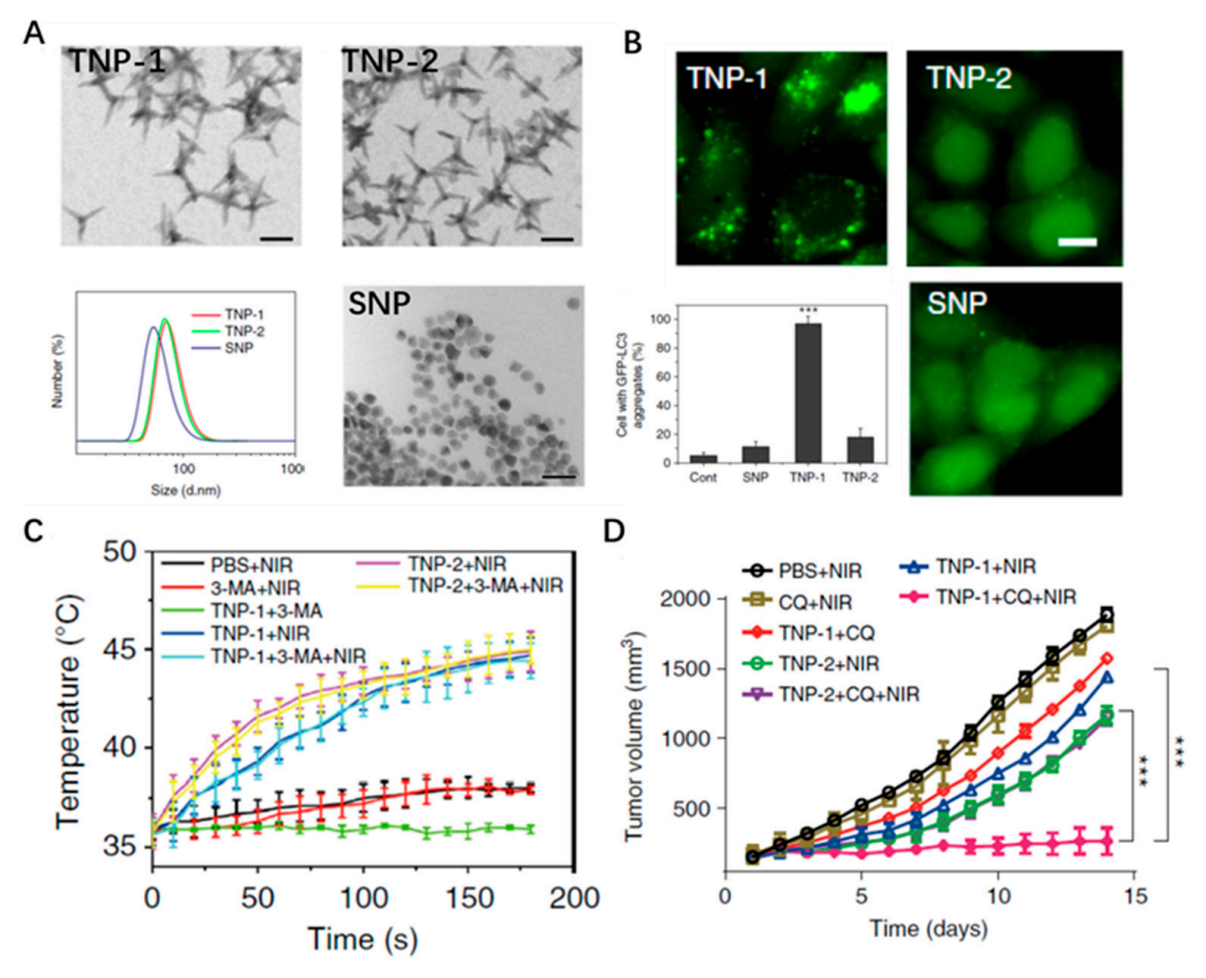
Enhanced PTT of TNP-1 for drug-resistant cancer in combination with autophagy inhibitors 3-MA or CQ. (**A**) TEM images of CuPd TNP-1, TNP-2, and SNP and size distribution of CuPd nanoparticles in aqueous solution as measured by dynamic light scattering (DLS). (**B**) Fluorescent microscopy images of EGFP-LC3/HeLa cells treated with PBS (control) or 10 μg mL^−1^ of CuPd nanoparticles for 24 h. Scale bar, 10 μm. The right panel shows the quantified results for the percentage of cells containing at least five EGFP-LC3 dots. Mean ± s.e.m. *n* = 5. *** *p* < 0.001. (**C**) The tumor-site temperature changes recorded during NIR irradiation for the various times, 24 h after the indicated PTT treatment. (**D**) Changes of mice in the various treatment groups during the 15-day therapeutic period. Doses used: TNP-1 or TNP-2, 1.5 mg kg^−1^; CQ, 25 mg kg^−1^; 3-MA, 100 μmol kg^−1^. Mean ± s.e.m. *n* = 5. *** *p* < 0.001. Student’s *t*-test (adapted from Reference [48] with permission from Nature Communications. Copyright 2018, Zhang, Y., Sha, R., Zhang, L. et al.).

Phototherapy has drawn increased attention in the field of cancer therapy due to its non-invasive and targeted cell-killing effects, which is ascribed to the inducing of hyperthermia (photothermal therapy) and production of highly toxic reactive oxygen species (ROS) (photodynamic therapy) with light stimulation. It is known that cytoprotective autophagy is imperatively induced in cancer cells as a result of the above-mentioned adverse cellular stress and might participate in resistance to phototherapy. Nevertheless, the therapeutic efficacy of PTT is still restricted by the indiscriminate heating. An ideal solution to overcome this limitation is chemo-PTT, which combines chemotherapy with photothermal therapy. However, there still remains giant challenges in the pro-survival autophagy elicited by chemotherapeutics and hyperthermia. For this question, we can attain an answer in Zhang’s proof of concept study [48], where they showed a sharp-tip structure of copper (Cu)-palladium (Pd) alloy tetrapod nanoparticles (TNP-1) capturing outstanding photothermal property. Compared to TNP-1, TNP-2 has a nearly identical morphology but with a different composition, which has the capacity for higher photothermal conversion efficiency but has no autophagy-inducing activity. Interestingly, TNP-1 can induce pro-survival autophagy in a shape- and composition-dependent manner. As shown in Figure 4, the combination with 3-methyl adenine or chloroquine has a remarkable synergistic effect on TNP-1-mediated PTT to achieve the same level of efficacy of TNP-2.

**Figure 5:**
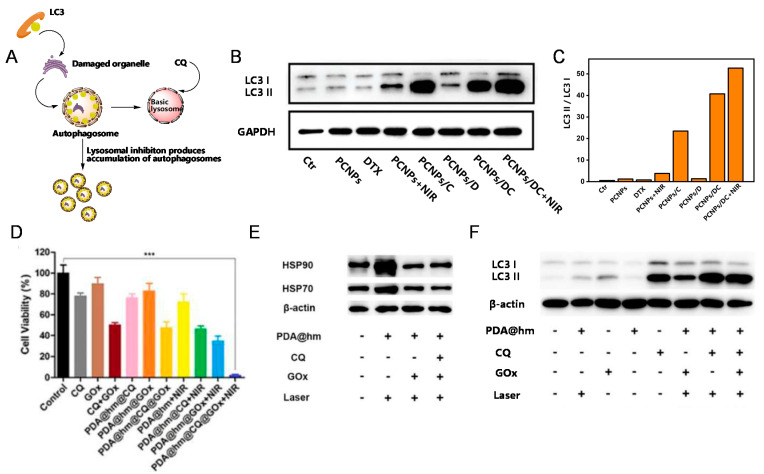
(**A**) The mechanisms of autophagy inhibition induced by CQ and relative LC3-mediated pathway. (**B**) Western blot analysis of LC3-I and LC3-II expression in MDA-MB-231 cells under different treatments. (**C**) Corresponding gray-scale analysis of the western blot analysis. (**D**) Augmented cell cytotoxicity by mild PTT in combination with glucose consumer GOx and autophagy inhibitor CQ in HepG-2 cells. *n* = 3; *** *p*< 0.001. (**E**) Representative expression of HSP90 and HSP70 after different treatments by western blot analysis. (**F**) Relative LC3-I and LC3-II expression in HepG-2 cells after different treatments. β-actin was used as an internal control.

Gold-based PTT [49] interacted synergistically with bortezomib and autophagy inhibition as the combined treatment to suppress the viability of U-87 MG cells, thereby suggesting NBP/TiO2 function as a promising anti-tumor agent. Inspired by these concerns, multifunctional nanoplatform incorporating multiple treatments have gained increased attention in recent studies. Rattle-structured polydopamine@mesoporous silica nanoparticles (PDA@hm@CQ@GOx) [50] and biomineralized nanocomposites (PCNPs/DC) [51] were designed by our group (Figure 5), which were loaded with autophagy inhibitor chloroquine (CQ). The effective inhibition of autophagy in cancer cells could be realized by blocking the lysosome and weakening the degradation of autolysosome by PCNPs/DC (Figure 5A–C). Notably, these functional nanoplatforms could sensitize PDA-based PTT through inhibiting autophagy via CQ. Moreover, PDA@hm@CQ@GOx could exhibit a significant enhancement of PTT through not only autophagy inhibition but also enhanced glucose depletion by GOx to suppress HSP70 and HSP90. PCNPs/DC would enhance DTX-mediated chemotherapy and PDA-mediated PTT through complementary autophagy inhibition (Figure 5D–F). However, there are challenges underlying common strategies for inhibiting autophagy, such as the hysteresis of small molecule inhibitors and instability of gene delivery. Hence, strikingly, an intriguing study has been conducted to develop a novel alternative strategy to realize autophagy inhibition.

PDT has emerged as an efficient therapeutic regimen that exerts a cytotoxic effect via ROS. Indeed, evidence arising from experiments involving the relationship between autophagy and PDT has indicated that pro-survival autophagy induced by PDT attenuates the efficacy of PDT as a cancer treatment. Wan’s group [52] designed an ATP-regulated ion transport nano-system (SQU@PCN) which provided the first demonstration on achieving autophagy inhibition through intelligent ion transport. SQU@PCN was disintegrated in response to abundant ATP in tumors and the incorporated SQU was then released. Accordingly, the SQU-medicated coupling transport of H^+^/Cl^−^ across the lysosomal membrane resulted in autophagy inhibition via increasing the lysosomal pH. Excellently, it can make up for the loss of PCN-based PDT efficiency and significantly enhance the efficacy of PDT. What’s more, SQU is involved in the dysregulation of chloride influx to deploy homeostatic perturbation therapy (HPT). Such a novel ideal strategy of ion transport nano-system provides a new direction for clinical cancer treatment research.

## 6. Combined with Cancer Sonodynamic Therapy

Similarly, ultrasound-based sonodynamic therapy (SDT) as an emerging alternative regimen is also one of the representative therapeutic modalities for noninvasive cancer treatment. ROS-mediated SDT has drawn widespread attention owing to its similar treatment paradigm to PDT [60]. Compared with PDT, SDT has the capacity to overcome the critical limitations of PDT, such as low tissue-penetration depth and phototoxicity. Despite its advantages, it was reported that there remains imperative challenges underlying the ROS-induced resistance of cancer cells. In the late stage of treatment, low-SDT dosage-induced autophagy was not enough to induce cell death, though it might have a pro-survival role through recycling nutrients and replenishing the energy supply for the tumor, as inspired by a study by Wan [49].

**Figure 6:**
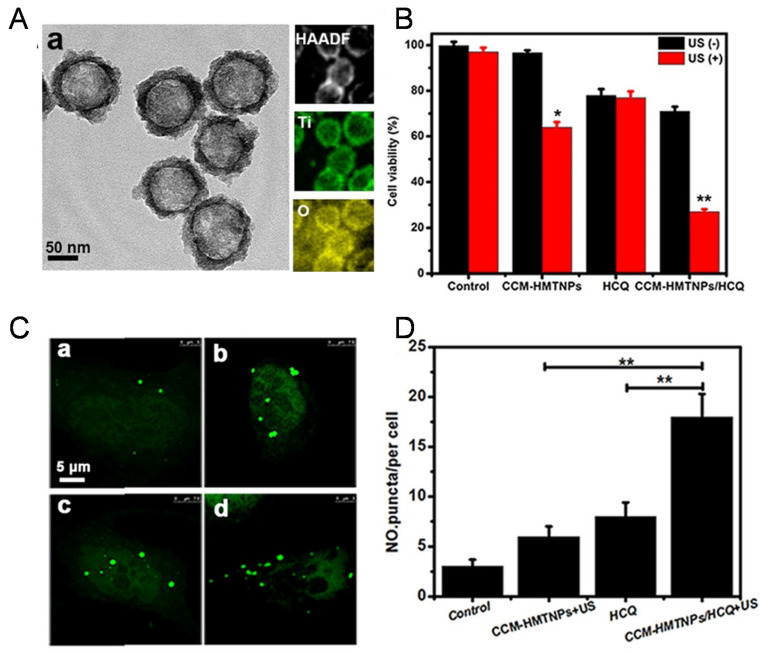
Enhanced SDT for therapeutic-resistant cancer by exploiting CCM-HMTNPs/HCQ. (**A**) TEM image (**left**) and the correspondent HAADF-STEM image (**right**) of HMTNPs, elemental mapping showing the distribution of Ti (green) and O (yellow). (**B**) Cell viability of MCF-7 cells with different treatments under US irradiation (1 W cm^−2^, 30 s). Note: the control group represents the cells treated with PBS. The data points represent mean ± S.D. (*n* = 6). The level of statistical significance is indicated when appropriate (* *p* < 0.05 versus control, ** *p* < 0.01 versus control). (**C**) Representative images and (**D**) quantification of eGFP-LC3-positive puncta of MCF-7 cells. Note: (**a**) Control group, (**b**) CCM-HMTNPs + US, (**c**) HCQ, and (**d**) CCM-HMTNPs/HCQ + US. (adapted from Reference [53] with permission from American Chemical Society. Copyright 2019, ACS Applied Materials & Interfaces).

From this viewpoint, Feng [53] developed a CCM biomimetic nanoplatform based on HMTNPs loaded with HCQ for optimizing sonodynamic therapy on breast cancer via autophagy inhibition. In response to US stimulus, HCQ was released and deacidified lysosome to block autophagic flux. There exists a prominent feature for HCQ which could alleviate tumor hypoxia by improving tumor perfusion and oxygenation to enhance the efficacy of oxygen-dependent SDT treatment. More importantly, CCM-HMTNPs/HCQ with a homologous targeting capacity could elevate the SDT sensitivity of MCF-7 cells by improving tumor oxygenation and blocking autophagic flux to abrogate the cells’ resistance to SDT (Figure 6).

## 7. Integrated Cancer Immunotherapy

Recently, it is highly desired to activate the host immune system against cancer, which is anticipated as a promising strategy. However, there remains unsatisfactory efficacy within current immunotherapeutic approaches such as vaccines and cytokines due to the high level of immune suppressive networks involved in cancer [61]. A couple of recent studies have focused on targeting the immune checkpoint blockade for treating tumors. In this sense, the PD-L1/PD-1 pathway [62,63] is considered the most valuable target, which is involved in dampening the immune response to protect tumor cells from immune destruction. PD-1 is highly expressed on the surface of tumor cells and can exhaust cytotoxic T-lymphocyte cells to protect tumor cells from immune destruction when it is engaged by PD-L1. Evidence arising from experiments indicated that PDT treatment could enhance the immune response. Yu et al. [19] synthesized the bovine serum albumin-Zinc phthalocyanine (ZnPc/BSA) nanoparticles-induced PDT treatment. Excitedly, the downregulation of PD-L1 was identified in response to the combination of the autophagy inhibitor 3-MA with PDT treatment, which opened the possibility of integrated therapy with autophagy inhibition and immunotherapy. Several clinical trials have aimed to block the PD-1/PD-L1 pathway to realize immunotherapy treatment, for instance, PD-1/PD-L1 inhibitors have demonstrated promising activity. However, current immune checkpoint blockade monotherapy cannot exert a satisfactory efficacy, which paves the way for integrated immunotherapy with multiple treatments. Ruan’s group [55] proposed a combination regime for glioma treatment based on functional gold nanoparticles (D&H-A-A&C)-enabled chemotherapy, autophagy inhibition, and the blockade of the PD-L1 immune checkpoint. In response to the overexpressed legumain, an enhanced accumulation of DOX and HCQ was observed. As a consequence, HCQ was proven to inhibit autophagy and re-sensitize glioma cells to DOX and inhibit the formation of vasculogenic mimicry. Moreover, D&H-A-A&C plus anti-PD-L1 antibody further enhanced anti-glioma effects due to the integrated cancer immunotherapy.

## 8. Conclusions

Albeit, except for the classical autophagy inhibitors such as CQ and 3-MA, delivery occurs via nano-carries for autophagy inhibition, and current studies are being conducted to integrate RNA interference with nanodrugs as a method that could be identified as a more temperate strategy to mitigate the low loading efficiency of small molecule inhibitors. Intrinsically, some nanomaterials such as iron oxide nanoparticles were reported to induce autophagy dysfunction by blocking autophagy flux, and intriguingly, an anion transport SQU-mediated nano-system exhibited the capacity to alkalize lysosome, as is the case with CQ. Above all, it will be of great importance to design novel functional nanotechnological platforms based on autophagy inhibition to increase the sensitivity of therapeutic strategies for tumor. Despite the seemingly promising nanodrugs in the context of tumor treatment, there are expansive, open issues to cope with. To date, it is unknown about the side effects of autophagy inhibition for use in combinatorial therapy based on nanotechnological platforms. Accordingly, it is a giant challenge and mission for both scientists and clinicians, to successfully translate preclinical knowledge of the nanoparticle-mediated autophagy inhibition strategy for tumor treatment into a clinical environment.

## Figures and Tables

**Table 1:** Summary of combinatorial therapy based on nanotechnological platforms-mediated complementary autophagy inhibition.

Autophagy Inhibition Mediated Nanoplatform	Combined Therapy	Strategy for Autophagy Inhibition	Reference
Self-assemble nano-prodrug platform siBec1@PPN	Chemotherapy	Beclin1 siRNA	[44]
A crosslinked redox-sensitive polypeptide micellar system	Chemotherapy	ATG7 siRNA	[45]
A multifunctional micelle vehicle	Chemotherapy	ATG7 siRNA	[46]
A carrier-free PTX nanocrystals (PNs)	Chemotherapy	MiR-101	[45]
Radiosensitizer based on gold nanoparticles (GNSs)	Radiotherapy	3-MA	[47]
Copper (Cu)-palladium (Pd) alloy tetrapod nanoparticles	Photothermal therapy	3-MA/CQ	[48]
Titania-coated gold nano-bipyramid (NBP/TiO_2_) nanostructures	Photothermal therapy	Nanomaterials	[49]
Rattle-structured polydopamine@mesoporous silica nanoparticles (PDA@hm@CQ@GOx)	Photothermal-Starvation therapy	HCQ	[50]
Biomineralized nanocomposites (PCNPs/DC)	Photothermal-Chemotherapy	HCQ	[51]
An ATP-regulated ion transport nano-system (SQU@PCN)	Photodynamic-Homeostatic perturbation therapy	Nanomaterials	[52]
CCM biomimetic nanoplatform based on HMTNPs	Sonodynamic therapy	HCQ	[53]
Bovine serum albumin-Zinc phthalocyanine (ZnPc/BSA) nanoparticles	Immuno-Photodynamic therapy	3-MA	[54]
Functional gold nanoparticles (D&H-A-A&C)	Immuno-Chemotherapy	HCQ	[55]

## Data Availability

The data presented in this study are available upon request from the cited author.
